# Ladder Polyphenylsilsesquioxanes and Their Niobium–Siloxane Composite as Coating Materials: Spectroscopy and Atomic Oxygen Resistance Study

**DOI:** 10.3390/polym15153299

**Published:** 2023-08-04

**Authors:** Ulyana S. Andropova, Rinat R. Aysin, Olga A. Serenko, Tatyana O. Ershova, Anton A. Anisimov, Vladimir N. Chernik

**Affiliations:** 1 A. N. Nesmeyanov Institute of Organoelement Compounds, Russian Academy of Sciences, 119991 Moscow, Russia; andropova@ispm.ru (U.S.A.); ershovatatyana_2995@mail.ru (T.O.E.); anisimov.ineos@gmail.com (A.A.A.); 2 D. V. Skobeltsyn Institute of Nuclear Physics, Lomonosov Moscow State University, 119234 Moscow, Russia; chernik@sinp.msu.ru

**Keywords:** ladder polyphenylsilsesquioxane, atomic oxygen resistance, transparency, hybrid particle, nanocomposite

## Abstract

In order to expand the range of materials that can be used in outer space and in development of small spacecraft, ladder polyphenylsilsesquioxanes with different molar weights and the Nb-siloxane composites based on them were studied. The properties of the polymer films were studied, including tests in an oxygen plasma flow. Both initial and filled ladder polymers feature extremely low erosion coefficients in the region of 10^–26^ cm^3^/atom O at a high fluence of atomic oxygen of 1.0 × 10^21^ atom O/cm^2^. Ladder polyphenylsilsesquioxane films irradiated with atomic oxygen (AO) retain their integrity, do not crack, and exhibit good optical properties, in particular, a high transmittance. The latter slightly decreases during AO exposure. The Nb-siloxane filling retains the AO resistance and slight decrease in optical transmission due to diffuse scattering on the formed Nb-[(SiO)_x_] nanoparticles. Ladder polyphenylsilsesquioxanes demonstrate their suitability for creating protective, optically transparent coatings for small spacecraft that are resistant to the erosive effects of incoming oxygen plasma.

## 1. Introduction

At present, remote sensing space systems based on small spacecraft (SSC) are widely used. The emergence of new materials, technologies and the development of electronics makes it possible to significantly reduce the weight, size and energy characteristics of small spacecraft and reduce their cost.

The near-satellite environment at altitudes of 200–800 km is aggressive with respect to polymeric materials and SSC coatings: high vacuum, ultraviolet radiation, temperature drops, charged particles, micrometeorites, atomic oxygen (AO), etc. [1,2,3,4]. Among these factors, the greatest danger for polymers used in the external elements of the SSC design is AO, whose incoming flow causes their erosion and degradation, which leads to a reduction in the service life of the SSC [5,6,7,8,9]. Research in areas such as the diversification of materials that can work in outer space and be used in the development of SSC is in line with global trends in the development of materials chemistry. 

Siloxane polymers are a class of non-carbon materials, whose erosion rate when exposed to AO is one to two orders of magnitude lower than that of organic polymers in a low-Earth orbit [7,10,11]. In addition to a lower erosion rate upon exposure to AO, siloxane polymers form a protective barrier layer of silicon dioxide, consisting of SiO_x_ fragments (where x ranges from 1.8 to 2.0) [11,12,13,14,15,16]. With constant exposure to AO, the inorganic surface layer prevents further degradation of the polymer underneath. However, this siloxane-to-silica surface transformation tends to create tensile stresses leading to coating cracking and loss of its integrity [10,11,12,13,14,15,16,17].

The traditional approach through the addition of fillers and modifiers makes it possible to partially solve this problem [16,17,18,19,20] that, in fact, prevents silicones from competing, for example, with polyimides capable of withstanding AO flow. Currently, silicones, due to their ability to form an inorganic protective layer when exposed to AO, play the role of a “second component” for polyimides. Thus, the application of silicones, including polyhedral oligomeric silsesquioxane, in the form of thin coatings on polyimide surfaces [18,19,20,21,22,23,24,25,26] or the introduction of -Si-O-Si- monomers, or blocks into the composition of polyimide macromolecules [27,28,29,30] sharply increases the resistance of these polymers to the negative factors of the space environment.

In the present paper, we have proposed that the above limitation of silicones can be overcome by resorting to a different strategy for improving these polymers, i.e., not adding additional components into siloxanes, but changing the molecular architecture of the siloxane chain by moving from a linear to a ladder structure.

The interest shown in ladder polymers is not accidental. It is ladder polymers that have the highest possible thermal stability among organic polymers, since their decomposition is associated with the breaking of at least two simple bonds in one cycle. The glass transition temperature of ladder polymers, as a rule, exceeds their decomposition temperature [31,32].

Ladder polyphenylsilsesquioxanes (L-PPSQs) are organosiloxanes with unique physicochemical properties: high thermal and radiation resistance, high refractive index, and solubility in organic solvents [31,32,33,34,35]. Films based on them have good dielectric properties and a high mechanical strength [34]. The widespread opinion that ladder-polymer-based materials have high strength but are brittle, which can lead to significant mechanical stresses and discontinuities in coatings based on them, was called into question by the authors of [36,37]. They developed a universal method for the synthesis of high-molecular L-PPSQs, which form elastic transparent films with high mechanical and thermal characteristics.

In this work, we studied the mechanical and optical properties of L-PPSQs with different molecular weights, as well as their resistance to atomic oxygen. Previously, we studied the effect of hybrid metal–siloxane nanoparticles formed as a result of sol–gel process from metal–siloxanes in the volume of a polyimide matrix [38,39,40]. It was shown that the valence of the central metal atom predetermines the content of silicate block in the shape-forming filler and, consequently, its AO-protective function. Among the investigated PI-filled films, the nanocomposite obtained using pentakis-(diethoxymethylsiloxy)niobium exhibited the best AO-resistant properties. Therefore, the latter was used as a filler in L-PPSQs to study the feasibility of further increasing the resistance of these polymers to AO-induced erosion.

## 2. Materials and Methods

### 2.1. Materials

Ladder polyphenylsilsesquioxanes (L-PPSQs) with different molecular weights: *M_w_* = 322 × 10^3^, *M_w_*/*M_n_* = 3.04 (L-PPSQ-322) and *M_w_* = 1060 × 10^3^, *M_w_*/*M_n_* = 2.99 (L-PPSQ-1060) were used. Polymers were obtained by condensation of *cis*-tetraphenylcyclotetrasiloxanetetraol (*cis*-tetraol), whose synthesis is described in paper [41], according to a known method [36] in ammonia medium. The molecular weight of the synthesized ladder polyphenylsilsesquioxane was determined by GPC using a chromatograph Shimadzu with RID-20A refractometer as a detector and analytical columns PSS SDV 10^5^ Å (size 300 × 8 mm). The eluent was THF. 

**Scheme 1:**
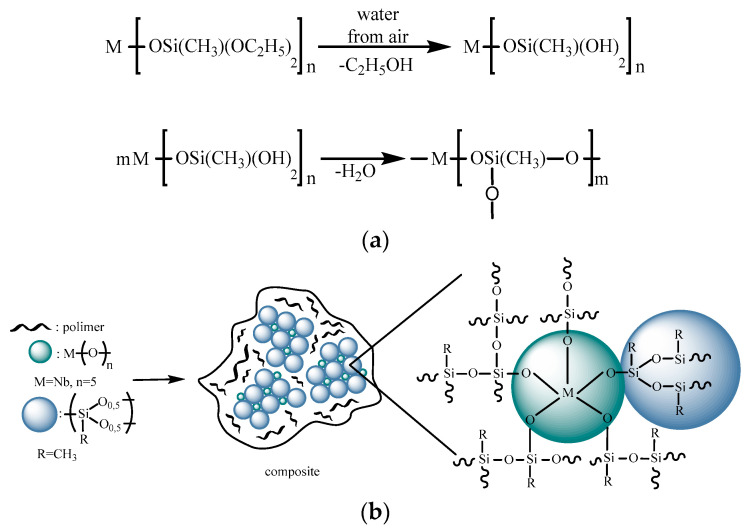
Assumed scheme of metalloalkoxysiloxane hydrolysis, condensation (**a**) and formation of polymer composite (**b**).

Pentakis-(diethoxymethylsiloxy)niobium was synthesized using the methods of [39] and used as the precursor of the composite dispersed phase. As a result of the precursor hydrolytic polycondensation, hybrid particles (Nb-[(SiO)_x_]) [38,39] are formed in the polymer. The formation of filled polymer by hydrolysis and condensation reactions, during which the metalloalkoxysiloxane precursor is transformed into a highly crosslinked material, are shown in Scheme 1.

It should be noted that we failed to determine the particle size in the matrices of ladder polyphenylsilsesquioxanes by SAXS, WAXS, and transmission electron microscopy due to the equal electron density of the particles and the matrix.

**Figure 1:**
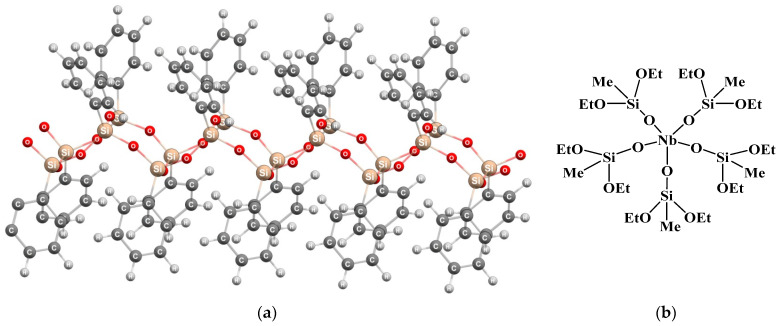
The structural fragment of ladder–like polyphenylsilsesquioxanes (L-PPSQs) (**a**) and structural formula of pentakis–(diethoxymethylsiloxy)niobium (**b**).

Figure 1 shows a fragment of the ladder polyphenylsilsesquioxane chain structure and the structural formulas of the precursor.

The polymer composition prepared on the basis of commercial α,ω-hydroxypolydimethylsiloxane “SKTN-D” (SAZI, Moscow, Russia) and pentakis-(diethoxymethylsiloxy)niobium (PDMS-Nb-[(SiO)_x_]) was used as a reference sample. The method for obtaining it is described earlier [39,40].

### 2.2. Sample Preparation

L-PPSQ-322 or L-PPSQ-1060 was dissolved in o-dichlorobenzene (5 wt.% solution), filtered and poured onto a glass substrate, which was gradually heated to 100 °C using a compact film-casting machine “MSK-AFA-IIID Automatic Thick Film Coater” (Shenyang, China). After complete removal of the solvent, the films were dried in a vacuum oven at 200 °C for 24 h.

In situ-filled films of L-PPSQs were obtained under similar conditions. The precursor concentration was 14 wt.% relative to the polymer, which corresponds to 8 wt.% of the resulting filler in the polymer. 

A precursor solution in toluene was added to 0.20 g of SKTN-D in 1 mL of anhydrous toluene at constant stirring under argon flow. The resulting solution was poured into a PTFE dish and dried at room temperature for 2 days. Next, the polymer film was thermally treated for 5 h in a drying chamber by constantly elevating the temperature from 50 to 200 °C. The concentration of the precursor that acts as a cross-linking agent [39,40] was 14 wt.%, as in the case of L-PPSQ.

### 2.3. Characterization

The mechanical properties (Table 1) were studied using an LLOYD Instruments LR5K Plus (UK). Test samples with a working size of 13 × 4 mm were used. The deformation rate was 60 mm/min. The film thickness (Table 1) was measured using a micrometer Etalon MK-25 (Moscow, Russia) with an accuracy of 4 μm.

UV-vis spectra in the region 220–900 nm with optical slit 2 nm and step 2 nm were registered using a Shimadzu UV-2600i (Kyoto, Japan) spectrophotometer equipped with an integrating sphere iSR-2600Plus. For all samples, transmission coefficient values (***τ***) were calculated with respect to the spectral range. The ***τ*** values for full transparence range 300–900 nm are presented and discussed in the main text. The ***τ_vis_*** values corresponded to the visible spectral range of 380–700 nm and ***τ***_D_ values calculated at the D-line of Na (589 nm) are presented in the Supplementary Materials (Table S1). The normalization of ***τ*** values to a thickness of 25 μm was performed using the Beer–Bouguer–Lambert law *A* = –*lg* ***τ***.

The FTIR transmission and ATR spectra in the range 400–4000 cm^–1^ with optical resolution 2 cm^–1^ were registered using a FTIR-spectrophotometers Bruker Tensor 37 (Mannheim, Germany) and Bruker Vertex 70 V (Mannheim, Germany) equipped with the diamond ATR accessory GladiATR. The intensity of ATR spectra was recounted to absorbance using the OPUS 7 program.

The surface morphology was studied by scanning electron microscopy (SEM). In this case, a JSM-6000PLUS scanning electron microscope (JEOL, Tokyo, Japan) was used with preliminary sputtering onto the surface of an electrically conductive coating.

### 2.4. Atomic-Oxygen Exposition

The testing experiments were carried out on a magnetoplasmodynamic gas pedal simulating low-Earth orbit conditions [42]. Film samples with a size of 20 × 20 mm were used. The facility consists of an evacuated vessel in which a plasma accelerator is placed. The vessel has a specimen holder and beam diagnostic equipment. Using vacuum pumping by cryogenic pumps with a rate of 5 m^3^/s, the vessel maintains a pressure of (0.5–2) × 10^–2^ Pa with plasma-supporting gas-oxygen requirements of 0.5 L Pa/s. The beam components are atoms, molecules and oxygen ions with a predominance of atomic ions. The power flux density absorbed by the specimen was 15 mW/cm^2^, which approximately corresponds to the heating of the specimen by solar radiation in space. Film samples 20 × 20 mm in size were used. The specimens were degaussed beforehand and held for 24 h at a temperature of 20 °C in a vacuum of 10^–4^ Pa.

Specimens were irradiated by an oxygen plasma beam from a plasma accelerator, simulating low-Earth orbit conditions. To ensure the same exposure, the analyzed specimens and the reference specimen were mounted on a rotating disk, placed normally in the plasma flow. The specimens’ masses were measured outside the evacuated vessel on an analytical microbalance HR-202i (AND, Tokyo, Japan) with a scale multiplier of 10^–5^ g, before and after each irradiation cycle with plasma flow. 

In the experiment, the effective fluence method was used to determine the intensity of AO exposure [43]. The equivalent AO fluence, *F* (O atoms cm^–2^), was determined by the change in weight of a reference sample (Kapton H polyimide film, DuPont, Wilmington, DE, USA) with an erosion yield of *E_K_* = 3 × 10^–24^ cm^3^/ atom:

(1) $$F=\frac{\Delta m_{K}}{S\rho_{K}E_{K}}$$

where Δ*m_K_* is the reference sample weight loss (g) during AO exposure, *S* is the exposed surface area (cm^2^) and *ρ_K_* is the density of the reference sample (1.42 g/cm^3^). The effective AO flux density in polyimide equivalents was (3–4) × 10^16^ atom/cm^2^ s. 

The erosion yield coefficient (*E_y_*, cm^3^ atom^−1^) of research samples is defined as the volume loss caused by one AO attack, calculated by Equation (2):

(2) $$E_{y}=\frac{\Delta m_{}}{S\rho_{}F}$$

where Δ*m* is the sample weight loss (g) during AO exposure, *S* is the exposed surface area (cm^2^), and *ρ* is the density of sample (for L-PPSQ *ρ* = 1.111 g/cm^3^ [44], SKTN-D siloxane rubber *ρ* = 0.980 g/cm^3^).

## 3. Results and Discussion

The samples of initial and filled polymers are flexible, high-strength films; moreover, these properties are preserved after AO irradiation. Figure S1 (in the Supplementary Materials) shows the stress–strain curves of the samples based on different matrix polymers and Nb-[(SiO)_x_]. Table 1 presents the mechanical characteristics after AO irradiation. As expected, due to a high molecular weight, the L-PPSQ-1060-based films exhibit better mechanical properties in comparison to those for the L-PPSQ-322-based films and close to those for L-PPSQs published earlier [38]. In particular, the modulus of elasticity of the AO-irradiated L-PPSQ-1060 film is 0.98 GPa, its strength and strain at break are 27 MPa and 6%, respectively. The data in Table 1 show that the in situ filling of the low- and high-molecular-weight L-PPSQs leads to different results. The tensile strength and modulus of elasticity for the L-PPSQ-1060-Nb-[(SiO)_x_] composite film increase against the L-PPSQ-322-Nb-[(SiO)_x_] film. Probably, the introduction of Nb-[(SiO)_x_] into the lower molecular polymer matrix L-PPSQ-322 leads to an embrittlement of the samples. Anyway, the modulus of elasticity, its strength, and strain at break are maintained at a good level after AO irradiation for all films studied.

**Figure 2:**
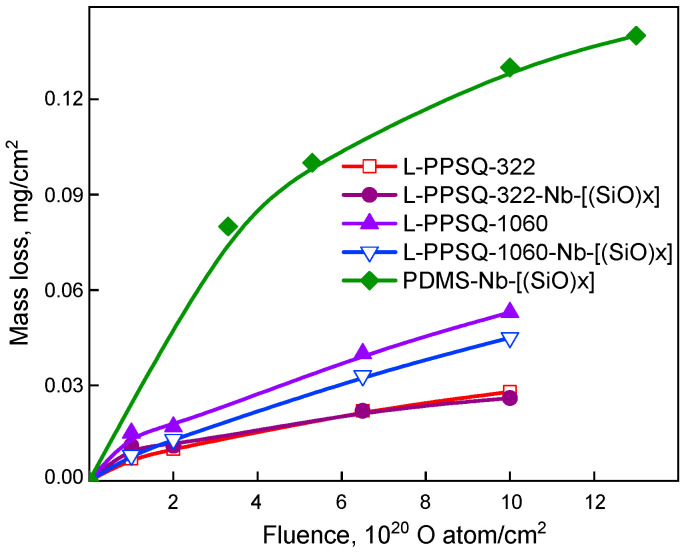
The mass loss dependence from AO fluence for the ladder–like polyphenylsilsesquioxane films, its composites and PDMS composite.

Figure 2 presents the dependences of the specific mass loss (Δ*m*/*S*, mg/cm^2^) of the initial L-PPSQs and the composites based on them. For comparison, the figure shows the dependence for PDMS-Nb-[(SiO)_x_]. The values of Δ*m*/*S* for both the initial and filled ladder polymers increase monotonically with increasing fluence (*F*), but they are small and do not exceed 0.05 mg/cm^2^ in the studied range of *F*. Also noteworthy is the absence of a significant effect of the filler in the polymer on the values of the specific weight loss of the composites. For example, the difference between Δ*m*/S values for unfilled and filled L-PPSQ-1060 at *F* = 1.0 × 10^21^ atom O/cm^2^ is 0.01 mg/cm^2^ and the dependences of Δ*m*/*S* on fluence for L-PPSQ-322-based samples coincide. The result obtained testifies to the equal resistance of the matrix and particles, as protective elements of the surface, to the action of AO. As can be seen from Figure 2 (the blue and violet curves), the polymer L-PPSQ-1060 and the composite based on it are less resistant to AO, compared with the samples of L-PPSQ-322 and L-PPSQ-322-Nb-[(SiO)_x_] (Figure 2, the red and purple curves). Dependences for the former run higher than for the latter. The AO resistance of PDMS-Nb-[(SiO)_x_] is worse than that of ladder polymers since the specific mass loss at *F* = 10 × 10^20^ atom O/cm^2^ is 2.5 times as high.

Table 2 shows the erosion coefficients (*E_y_*) of the studied films at different fluences and the reference samples. The *E_y_* values of ladder polymers are two orders of magnitude and one order of magnitude lower than those of the Kapton and PDMS-Nb-[(SiO)_x_]. Thus, the L-PPSQs exhibit very high performance with respect to AO resistance, which is comparable with the best polyimide composites.

**Figure 3:**
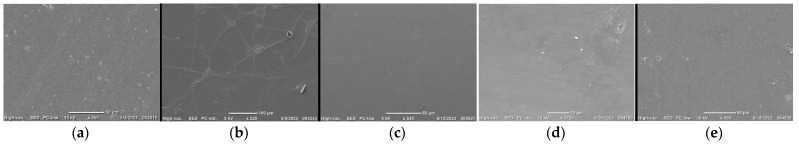
SEM images of the surface for the AO exposed films: L-PPSQ-322 (**a**), L-PPSQ-1060 (**b**), PDMS-Nb-[(SiO)_x_] (**c**), L-PPSQ-322-Nb-[(SiO)_x_] (**d**), and L-PPSQ-1060-Nb-[(SiO)_x_] (**e**) at *F* = 1.0 × 10^21^ atom O/cm^2^.

Figure 3 presents typical photographs of the surface of the AO-irradiated samples. The surface of the ladder polymer L-PPSQ-1060 and L-PPSQ-322 after exposure to AO does not contain defects, while cracks have appeared on the surface of the irradiated PDMS-Nb-[(SiO)_x_]. This is expected since it was previously known that siloxane coatings cracked upon exposure to a high atomic-oxygen effect [39] due to the resultant conversion to silicon dioxide, which induced tensile stress on the surface of the coating, whereas the introduction of Nb-siloxane nanoparticles is unable to counteract this. At the same time, there are micron-sized particles on the surface of composites based on L-PPSQ filled with Nb-[(SiO)x]; however, they do not damage the surface after AO irradiation (Figure 3d,e).

Thus, ladder polyphenylsilsesquioxanes are resistant to AO impact, feature low erosion coefficients, namely 2.5 × 10^–26^ cm^3^/atom O (L-PPSQ-322) and 4.8 × 10^–26^ cm^3^/atom O (L-PPSQ-1060) at AO fluence *F* = 1.0 × 10^21^ atom O/cm^2^. The effect of AO does not lead to surface discontinuity or cracking of the films of these polymers. Ladder polyphenylsilsesquioxanes can be classified as self-sufficient polymers that do not require the introduction of a filler as an additional protective element that can increase the resistance of the material against the destructive effects of AO.

**Figure 4:**
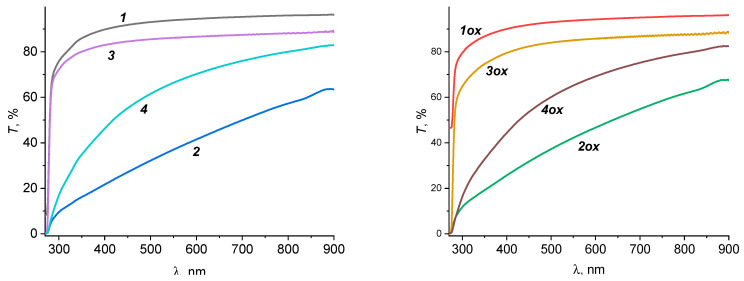
The normalized UV-vis transmission spectra for initial films (***1****–**4***) and AO-exposed films (***1ox****–**4ox***) of the following samples: L-PPSQ-322 (***1***), L-PPSQ-322-Nb-[(SiO)_x_] (***2***), L-PPSQ-1060 (***3***), and L-PPSQ-1060-Nb-[(SiO)_x_] (***4***).

**Figure 5:**
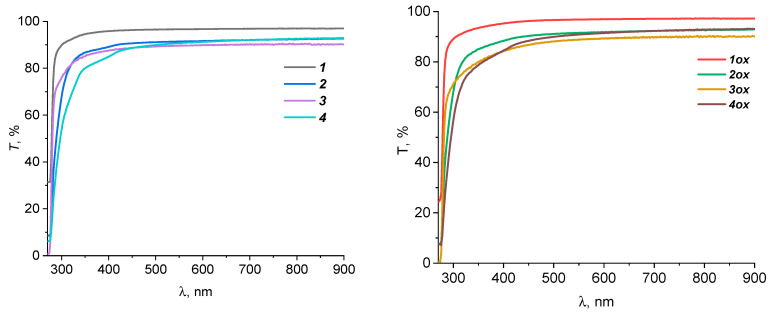
The normalized UV-vis transmission spectra registered using an integration sphere for initial films (***1****–**4***) and AO-exposed films (***1ox****–**4ox***) of the following samples: L-PPSQ-322 (***1***), L-PPSQ-322-Nb-[(SiO)_x_] (***2***), L-PPSQ-1060 (***3***), and L-PPSQ-1060-Nb-[(SiO)_x_] (***4***).

**Figure 6:**
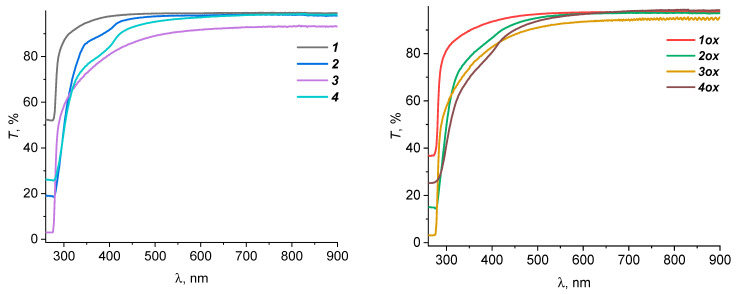
The normalized UV-vis diffuse-reflectance spectra for initial films (***1****–**4***) and AO-exposed films (***1ox****–**4ox***) of the following samples: L-PPSQ-322 (***1***), L-PPSQ-322-Nb-[(SiO)_x_] (***2***), L-PPSQ-1060 (***3***), and L-PPSQ-1060-Nb-[(SiO)_x_] (***4***).

The optical properties of the initial and AO-irradiated sample films based on ladder polyphenylsilsesquioxanes were characterized using absorption spectra, which were recorded in three ways. Figure 4 presents the standard spectra recorded in the transmission mode, based on which transmittance values ***τ****^TS^* were calculated (see Table 3). The use of an integrating sphere in the transmission mode makes it possible to collect diffuse scattered light (Figure 5) and thereby exclude the effect of light-beam scattering from the spectrum. Based on the spectra in Figure 5, ***τ****^DT^* values were calculated. The difference (Δ***τ***) between the ***τ****^TS^* and ***τ****^DT^* coefficients was used as an averaged characteristic to estimate the scattering degree of the light passing through the film. To identify weak absorption bands of Nb-[(SiO)_x_] particles in the composition, absorption spectra were recorded in the diffuse reflection mode (Figure 6) using an integrating sphere relative to the BaSO_4_ standard.

The spectra obtained (Figure 4) make it possible to conclude that films of pure ladder silsesquioxanes L-PPSQ-322 and L-PPSQ-1060 demonstrate a high degree of transmission in the range of 300–900 nm, while absorption in the ultraviolet region below 300 nm is due to the presence of lateral Ph-substituents. The investigated films of initial ladder polymers also have a high degree of transparency, which is numerically expressed in a small Δ***τ*** value (Table 2). Composite films, both L-PPSQ-322-Nb-[(SiO)_x_] and L-PPSQ-1060-Nb-[(SiO)_x_], scatter light to a large extent, which manifests itself as a slope of the baseline (Figure 4) and an increase in the value of Δ***τ*** by an order of magnitude.

A comparison of the spectra for L-PPSQ-322 and a composite based on it in different registration modes shows that the Nb-[(SiO)_x_] filler has its own weak absorption band of ~385 nm, which is more clearly seen in the diffuse reflection spectra (Figure 6). Obviously, the reason for the increase in diffuse scattering is the presence of particles in the polymer. Since in the spectra of L-PPSQ-322-Nb-[(SiO)_x_] and L-PPSQ-1060-Nb-[(SiO)_x_] composites (Figure 5), the slopes of the base lines differ, i.e., light scattering is different, it can be assumed that the particle size in the L-PPSQ-322-Nb-[(SiO)_x_] composite is somewhat larger.

AO irradiation of the films slightly changes the absorption coefficients, which can be seen when comparing the spectra of the original and irradiated films (Figure 4). For L-PPSQ-1060 and L-PPSQ-1060-Nb-[(SiO)_x_] films, the ***τ*** values decrease by no more than 0.01–0.02, and for L-PPSQ-322 and L-PPSQ-322-Nb-[(SiO)_x_], by 0.02–0.05. When the changes are small, the degree of scattering (Δ***τ***) also changes just slightly, since the treatment of the films with atomic oxygen mainly affects the surface.

**Figure 7:**
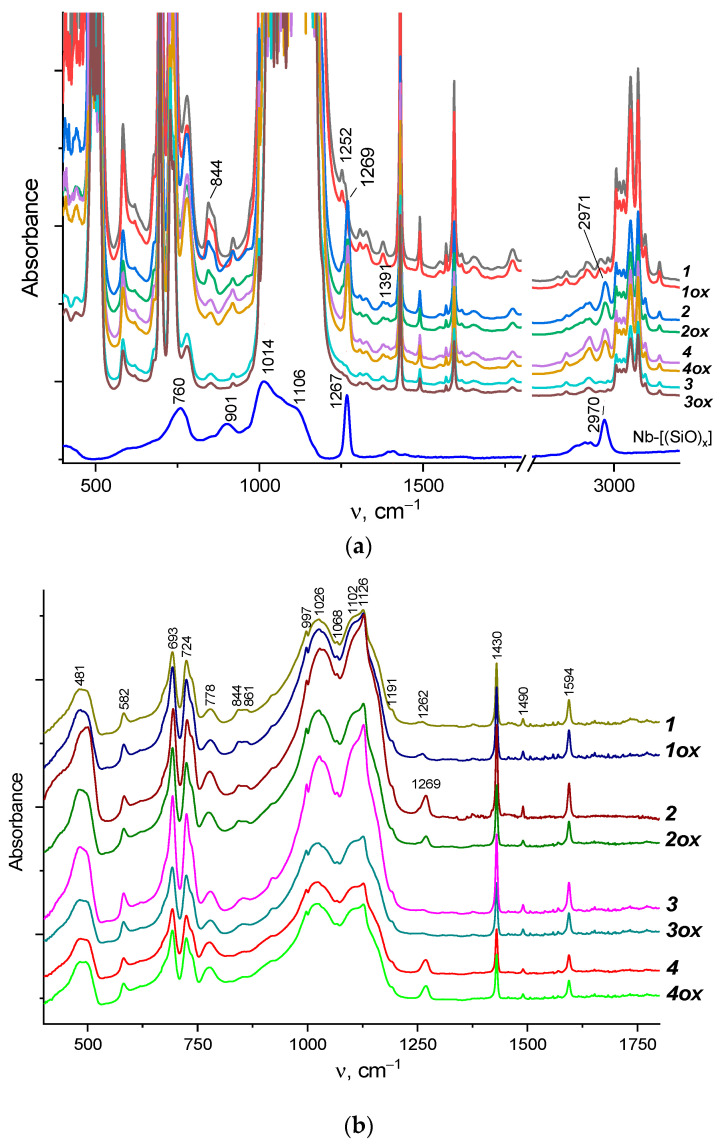
FTIR transmission spectra (**a**) and ATR FTIR spectra (**b**) for initial films (***1***–***4***) and AO-exposed films (***1ox***–***4ox***) of the following samples: L-PPSQ-322 (***1***), L-PPSQ-322-Nb-[(SiO)_x_] (***2***), L-PPSQ-1060 (***3***), and L-PPSQ-1060-Nb-[(SiO)_x_] (***4***).

The obtained FTIR spectra of the initial and AO-irradiated films (Figure 7) show a set of bands corresponding to the vibrations of the lateral Ph groups (the most typical bands are 693, 724 (out-of-plane δCH), 1430, 1594 cm^–1^ (aromatic C=C stretchings), and aromatic C–H stretchings > 3000 cm^–1^). The most intense broad bands at ~1020 and ~1100 cm^–1^ are related to ν^as^(Si-O-Si); their shape and position are typical of ladder polyphenylsilsesquioxanes [45]. The above ν^as^(Si–O–Si) and Ph bands go off scale in the transmission spectra (Figure 7a) due to the large thickness of the analyzed films. Attention is drawn to the manifestation of terminal SiMe_3_, for which bands are observed at 844 and 1252 cm^–1^, corresponding to the δ(Si–C) and δ(CH_3_) vibrations, respectively. The presence of particles in the composite films, including after exposure to AO, can be identified by the presence of the Me group, for which pronounced δ(CH_3_) 1269 cm^–1^ and ν^as^(CH_3_) 2971 cm^–1^ IR bands are observed (Figure 7a). At the same time, vibrations of OEt groups (first of all, deformational δ(CH) 1300–1450 cm^–1^) are not observed, which indicates their complete consumption at the first stage of the precursor sol–gel reaction [38,39]. Since the complex contour of the ν^as^(Si–O–i) bands is almost the same for unfilled films of ladder polymers and the corresponding filled films at 1000–1200 cm^−1^, we can assume that no incorporation of niobium alkoxysiloxane into the structure of the ladder polyphenylsilsesquioxanes is taking place. 

The effect of atomic oxygen can be traced by comparing the ATR-IR spectra (Figure 7b), since they are recorded from the film’s surface. It turned out that the ATR-IR spectrum of the untreated film almost completely coincides with the spectrum of the treated film, in contrast, for example, to a polyimide film or organically soluble polyimide-based nanocomposites [38]. This indicates the absence of structural changes or destructive processes in ladder polymers or nanocomposites based on them. In the transmission spectra of the AO-irradiated films, no additional bands are observed in the region of 1600–1800 cm^–1^ that could be attributed to carbonyl vibrations. This fact suggests that no partial oxidation with the formation of organic products during the oxidation/destruction of Ph groups occurs either on the surface or in the bulk of the film.

## 4. Conclusions

We studied the resistance to atomic oxygen of ladder polyphenylsilsesquioxanes (L-PPSQ) with molecular weights *M_w_* = 322 × 10^3^ and 1060 × 10^3^, as well as composites based on them. Both initial and filled ladder polymers exhibit low erosion coefficients in the region of 10^–26^ cm^3^/atom O at a fluence of atomic oxygen (AO) of 1.0 × 10^21^ atom O/cm^2^. L-PPSQ exhibits a high AO resistance (10^–26^ cm^3^/atom O) in comparison to the well-known polyimide Kapton (10^–26^ cm^3^/atom O) [7] and its composites (up to 10^–26^) [38] cm^3^/atom O, which was rationalized by the highly stable ladder structure of L-PPSQ. When exposed to an oxygen plasma flow, the films retain their integrity and do not crack, i.e., they are free from the limitation typical of linear siloxanes. Both unirradiated and irradiated ladder polyphenylsilsesquioxane films retain a high transparency. Exposure to high AO fluence does not lead to oxidation or degradation of both polymers and composites based on them. Filling in situ with pentakis-(diethoxymethylsiloxy)niobium as a filler precursor does not change the AO resistance of the polymers, but at the same time increases the diffuse scattering of filled films, reducing their transparency. Therefore L-PPSQ polymers are suitable for creating protective, optically transparent coatings for small spacecraft, and do not require any addition of fillers to increase the resistance of the material to the destructive effect of AO.

## Figures and Tables

**Table 1:** The thickness and mechanical properties for the ladder–like polyphenylsilsesquioxane films and its composites after the AO irradiation.

Sample	σ_p_, MPA	ε_p_, %	E, MPA	Film Thickness, μm
L-PPSQ-322	26 ± 2	7.0 ± 1.0	930 ± 56	120
L-PPSQ-322-Nb-[(SiO)_x_]	18 ± 1	2.5 ± 0.2	900 ± 54	38
L-PPSQ-1060	27 ± 2	6.0 ± 0.4	980 ± 59	50
L-PPSQ-1060-Nb-[(SiO)_x_]	40 ± 2	6.0 ± 0.4	1250 ± 75	27

**Table 2:** The erosion yield values (cm^3^/atom O) for the ladder–like polyphenylsilsesquioxane films, its composites and PDMS composite at different fluence values *F*.

Sample	at *F* = 6.5 × 10^20^ Atom O/cm^2^	at *F* = 10.0 × 10^20^ Atom O/cm^2^
Kapton (reference) [7]	3 × 10^–24^	3 × 10^–24^
L-PPSQ-322	3.0 × 10^–26^	2.5 × 10^–26^
L-PPSQ-322-Nb-[(SiO)_x_]	3.0 × 10^–26^	2.4 × 10^–26^
L-PPSQ-1060	5.5 × 10^–26^	4.8 × 10^–26^
L-PPSQ-1060-Nb-[(SiO)_x_]	4.6 × 10^–26^	4.0 × 10^–26^
PDMS-Nb-[(SiO)_x_]	1.7 × 10^–25^	1.3 × 10^–25^
PI- Nb-[(SiO)_x_] [38]	2.1 × 10^–26^	1.7 × 10^–26^
PMDA−ODA/HBPSi [27]	5.38 × 10^–25^	
POSS PI [29]	6.6 × 10^–26^	
PI-30 [26]	1.1 × 10^–25^	

**Table 3:** Transmission coefficient ***τ*** (300–900 nm) values for the polymer film studied before and after AO exposure.

Sample	*τ^TS^*		*τ^DT^*		Δ*τ*	
	Before AO Exposure	After AO Exposure	Before AO Exposure	After AO Exposure	Before AO Exposure	After AO Exposure
L-PPSQ-322	0.98	0.98	0.99	0.99	0.01	0.01
L-PPSQ-322-Nb-[(SiO)_x_]	0.59	0.63	0.95	0.94	0.36	0.31
L-PPSQ-1060	0.86	0.84	0.89	0.87	0.03	0.03
PPSQ-1060-Nb-[(SiO)_x_]	0.79	0.79	0.94	0.94	0.15	0.15
PDMS-Nb-[(SiO)_x_]	0.92	–	0.90	–	0.02	–

All ***τ*** values are normalized to a thickness of 25 μm. Δ***τ*** = ***τ****^DT^ – **τ**^TS^*^.^

## Data Availability

The data presented in this study are available on request from the corresponding author.

## Supplementary Materials

The following supporting information can be downloaded at https://www.mdpi.com/article/10.3390/polym15153299/s1. Table S1: Transmission coefficients ***τ****_vis_* and ***τ***_D_ normalized to thickness 25 μm. Figure S1: The stress-strain curves of the samples studied after AO irradiation.
