# Supplementary material for: Ladder Polyphenylsilsesquioxanes and Their Niobium–Siloxane Composite as Coating Materials: Spectroscopy and Atomic Oxygen Resistance Study
Source: Polymers (Basel). 2023 Aug 4;15(15):3299. doi: 10.3390/polym15153299 (PMC10422322; doi:10.3390/polym15153299)
Supplement: Supplementary file 1 [file polymers-15-03299-s001.zip › polymers-2508219-supplementary.pdf]

Supplementary Materials

Ladder Polyphenylsilsesquioxanes and Their Niobium–Siloxane Composite as Coating Materials: Spectroscopy and Atomic Oxygen Resistance Study

Ulyana S. Andropova [1], Rinat R. Aysin [1,*], Olga A. Serenko [1,*], Tatyana O. Ershova [1], Anton A. Anisimov [1] and Vladimir N. Chernik [2]

[1] A. N. Nesmeyanov Institute of Organoelement Compounds, Russian Academy of Sciences, 119991 Moscow, Russia; andropova@ispm.ru (U.S.A.); ershovatatyana_2995@mail.ru (T.O.E.); anisimov.ineos@gmail.com (A.A.A.)

[2] D. V. Skobeltsyn Institute of Nuclear Physics, Lomonosov Moscow State University, 119234 Moscow, Russia; chernik@sinp.msu.ru

* Correspondence: aysin@ineos.ac.ru (R.R.A.); o_serenko@ineos.ac.ru (O.A.S.)

Table S1. Transmission coefficients τ_{vis} and τ_D normalized to thickness 25 µm.

Sample	τ_{vis} (380-700 nm)			τ_D (589 nm)		
	τ_1^{TS}	τ_1^{DT}	τ_1^{DR}	τ_D^{TS}	τ_D^{DT}	τ_D^{DR}
L-PPSQ-322	0.95	0.99	0.99	0.98	0.99	0.99
L-PPSQ-322 [**ox**]	0.96	0.99	0.97	0.98	0.99	0.97
L-PPSQ-322-Nb-[$(SiO)_x$]	0.34	0.95	0.97	0.61	0.95	0.98
L-PPSQ-322-Nb-[$(SiO)_x$] [**ox**]	0.38	0.95	0.94	0.65	0.95	0.97
L-PPSQ-1060	0.78	0.89	0.89	0.87	0.90	0.92
L-PPSQ-1060 [**ox**]	0.73	0.88	0.91	0.86	0.89	0.93
L-PPSQ-1060-Nb-[$(SiO)_x$]	0.55	0.95	0.95	0.83	0.95	0.97
L-PPSQ-1060-Nb-[$(SiO)_x$] [**ox**]	0.54	0.95	0.3	0.83	0.96	0.97
PDMS-Nb-[$(SiO)_x$]	0.90	0.93	0.93	0.93	0.99	0.97

The "[**ox**]*" index denotes the samples exposed by atomic oxygen.*

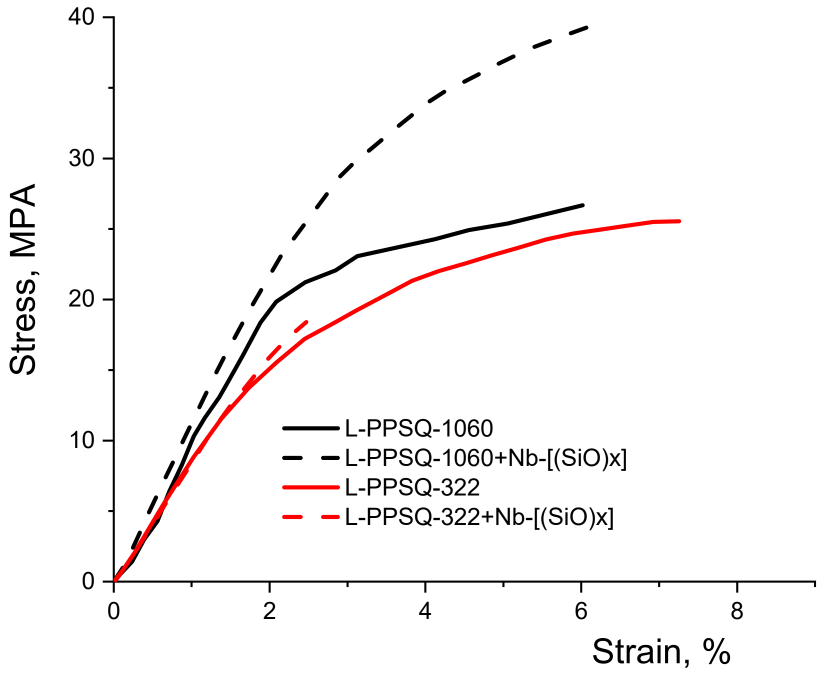

Figure S1. The stress - strain curves of the samples studied after AO irradiation.
